# Detection of *ESR1* Mutations Based on Liquid Biopsy in Estrogen Receptor-Positive Metastatic Breast Cancer: Clinical Impacts and Prospects

**DOI:** 10.3389/fonc.2020.587671

**Published:** 2020-12-15

**Authors:** Hao Liao, Wenfa Huang, Wendi Pei, Huiping Li

**Affiliations:** ^1^ Key Laboratory of Carcinogenesis and Translational Research (Ministry of Education/Beijing), Department of Breast Oncology, Peking University Cancer Hospital and Institute, Beijing, China; ^2^ Department of Hematology-Oncology, International Cancer Center, Shenzhen University General Hospital, Shenzhen University Health Science Center, Shenzhen, China; ^3^ Center for Reproductive Medicine, Department of Obstetrics and Gynecology, Beijing Key Laboratory of Reproductive Endocrinology and Assisted Reproductive Technology and Key Laboratory of Assisted Reproduction, Ministry of Education, Peking University Third Hospital, Beijing, China

**Keywords:** endocrine therapy, *ESR1*, estrogen receptor-positive metastatic breast cancer, liquid biopsy, sub-clone

## Abstract

Endocrine therapy is the main treatment option for estrogen receptor-positive (ER+) breast cancer (BC). Compared with other clinical subtypes, ER+ BC patients usually have a more favorable prognosis. However, almost all ER+ BCpatients develop endocrine resistance and disease progression eventually. A large number of studies based on liquid biopsy suggest that *ESR1* mutations may play a key role in this process. For patients with ER+ metastatic BC (MBC), *ESR1* is an important prognostic factor and may associate with the resistance to endocrine therapy, like aromatase inhibitors. The advances of sequencing technologies allow us to conduct longitudinal monitoring of disease and unveil the clinical implications of each *ESR1* sub-clone in ER+ MBC. Moreover, since the *ESR1*-related endocrine resistance has not been fully addressed by existing agents, more potent cornerstone drugs should be developed as soon as possible. Herein, we reviewed the recent progress of detecting *ESR1* mutations based on liquid biopsy and different sequencing technologies in ER+ MBC and discussed its clinical impacts and prospects.

## Introduction

Endocrine therapy (ET) is the main treatment option for estrogen receptor-positive (ER+) breast cancer (BC), which accounts for 70% of all diagnosed cases (1, 2). Three main ET strategies, including aromatase inhibitors (AIs), selective ER modulators (SERMs), and selective ER down-regulators (SERDs), are available in targeting the estrogen pathway (3). With the use of these agents, the 5-year survival rate of ER+ BC patients has been greatly improved since 1990 (4). However, endocrine resistance remains a major clinical challenge. Almost all ER+ BC patients develop endocrine resistance and disease progression eventually, suggesting the need to develop more potent drugs, as well as technologies for predicting and longitudinally monitoring the ET resistance (5, 6). The liquid biopsy provides a noninvasive route of sample collection for cancer patients. Using cell-free DNA (cfDNA), circulating tumor DNA (ctDNA), and circulating tumor cells (CTCs), liquid biopsy has been widely utilized in the research of various tumors (7–10).

*ESR1*, the gene that encodes ERα and mediates the biological effects of estrogen, is associated with the incidence of ER+ BC (11, 12). Compared with the *ESR1* mutations frequency of <5% in primary BC from the results of Cancer Genome Atlas project, Jeselsohn et al. reported a higher incidence of 12% (9/76) in advanced ER+ disease (13, 14). Allouchery et al. found that more hormone receptor-positive (HR+) BC patients had circulating *ESR1* mutations at progression than those at first relapse (33%, 7/21 vs. 5.3%, 2/38) (15). Compared with patients at early treatment lines, metastatic patients at late treatment lines had a higher prevalence of circulating *ESR1* mutations (16, 17). All the work mentioned above essentially underline the importance of detecting circulating *ESR1* mutations in metastatic settings. In recent years, the sequencing technology has developed rapidly from Polymerase Chain Reaction (PCR) to Next-Generation Sequencing (NGS), which allows efficient and accurate detection of genomic alterations in cancer and accelerates the process of precision medicine (18, 19). Herein, we summarized recent progress of detecting *ESR1* mutations based on liquid biopsy and different sequencing technologies in ER+ metastatic BC (MBC) and discussed its clinical implications and prospects.

## ERα Structure and Signaling

The ERα is a member of nuclear HR superfamily and acts as a ligand-activated transcription factor (20). The structure of ERα consists of two activating function domains (AF1/2), a DNA-binding domain, a hinge domain, and a ligand-binding domain (LBD). The most studied domain of ERα, LBD, has 12 α-helices (helix 1-helix 12) that are mostly linked by loop regions (21).

**Figure 1:**
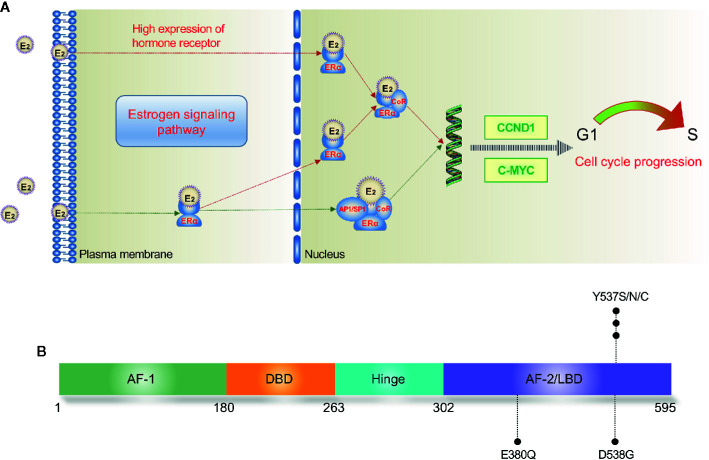
Estrogen signaling pathway **(A)** and major mutation sites of *ESR1* **(B)**. In the nucleus, E2 directly mediates the transcription by binding to the ERE of target genes; E2 also binds with transcription factors such as AP1 and SP1, leading to indirect regulation of DNA transcription. E2, estradiol; ERE, estrogen response element; ERα, estrogen receptor α; AF, activation function; DBD, DNA binding domain; LBD, ligand binding domain.

The ERα is involved in both genomic and non-genomic signaling. In the nucleus, estradiol binds to the LBD of ERα, leading to a conformational change of helix 12. Then, the conjugate of ERα and estradiol binds to the estrogen responsive element (ERE) of ERα-target genes, resulting in the recruitment of coregulatory proteins (22). The activating ER pathway drives the cell from G1 phase to S phase and causes cell proliferation consequently (**Figure 1A**). Reversely, binding to an anti-estrogen agent like tamoxifen (TAM) will prevent the helix 12 of LBD from forming an active AF-2 conformation (21). In addition, the ERα can interact with several other transcription factors such as NF-kB and AP-1 to indirectly regulate DNA transcription activity in an ERE-independent way (23, 24). At the plasma membrane, ERα also interacts with some growth factor receptors (GFRs) to achieve a non-genomic or transcription-independent function, including insulin growth factor 1 receptor (*IGF1R*), human epidermal growth factor receptor 2 (HER2), and fibroblast growth factor receptor (*FGFR*) (25).

## Overview of *ESR1* Mutations in MBC

The most commonly detected *ESR1* point mutations locate in codons 380, 537, and 538 within the LBD (19, 26) (**Figure 1B**). Several large-scale clinical trials have investigated the prevalence of ctDNA *ESR1* mutations in ER+ MBC patients (27–30). In a secondary-analysis to the BOLERO-2 study, *ESR1* D538G and Y537S mutations were found in 28.8% and 13.3% of the samples, respectively (27). The PALOMA-3 study reported an *ESR1* mutation rate of 25.3%, while both the SoFEA study and the FERGI study found relatively higher *ESR1* mutation rates (39.1% and 37%, respectively) in ER+ MBC patients (28–30). As shown in **Table 1**, four studies (BOLERO-2, SoFEA, PALOMA-3, and FERGI) revealed a high prevalence of *ESR1* mutations in second and higher treatment line, with a total mutation rate of 32% (684/2136), while one study (MONALEESA-2) evaluated the *ESR1* mutations in first-line treatment and reported a low prevalence of *ESR1* mutations (4%). Our previous work revealed an *ESR1* mutation rate of 24.7% (169/685) in the HR+ MBC patients (19). And this proportion seemed to be higher (30%–40%) in ER+ HER2- MBC patients who had resistance to ET, especially to AIs (31).

**Table 1:** Large-scale clinical trials of recent years detecting *ESR1* mutations in metastatic breast cancer (MBC).

Treatment line	Research	Year	Study type	Patient number	Prevalence of *ESR1* mutations
Second and higher-line exemestane + everolimus vs. exemestane + placebo	BOLERO-2 (NCT00863655)	2012	Phase-3 study	724	28.8%
Second-line fulvestrant + anastrozole or placebo vs. exemestane alone	SoFEA (NCT00253422)	2013	Phase-3 study	723	39%
Second and higher-line palbociclib + fulvestrant vs. fulvestrant + placebo	PALOMA-3 (NCT01942135)	2015	Phase-3 study	521	25.3%
Second and higher-line pictilisib + fulvestrant vs. fulvestrant + placebo	FERGI (NCT01437566)	2016	Phase-2 study	168	37%
First-line ribociclib + letrozole vs. letrozole + placebo	MONALEESA-2 (NCT01958021)	2016	Phase-3 study	668	4%

These studies detected ESR1 mutations in MBC patients. Most of them (4/5) revealed a high prevalence of ESR1 mutations in second and higher treatment line, with a total mutation rate of 32% (684/2136), while the MONALEESA-2 study evaluated the ESR1 mutations in first-line treatment and reported a low prevalence of ESR1 mutations (4%).

## Endocrine Resistance Associated With *ESR1*

The endocrine therapeutic approaches including AIs, SERMs (preferably TAM), and SERDs (preferably FUL) have become the backbone of treatment for ER+ MBC (2, 3). AIs can specifically inactivate aromatase and reduce the level of estrogen in the blood to achieve the purpose of treating BC (32). According to the chemical structure, AIs have been divided into steroidal AIs (exemestane and formestane) and non-steroidal AIs (aminoglutethimide, letrozole, and anastrozole). Both SERMs and SERDs block the function of ER. The difference between them is that SERMs have mixed agonistic/antagonistic capacities and compete with the estrogen ligand, while SERDs possess exclusive antagonistic activity and induce ER protein degradation (33). For post-menopausal ER+ HER2- MBC patients who have not received any anti-estrogen therapy, AIs, TAM, and FUL are reasonable choices, while for those who have failed anti-estrogen therapy, AIs are the first choice. For pre-menopausal ER+ HER2- MBC patients, the first-line treatment could be TAM alone, AIs plus ovarian suppression, or appropriate ET with CDK4/6 inhibitors like palbociclib (2). Nevertheless, the problem of endocrine resistance may arise due to the selective pressure of therapy (34). Current data indicate that the presence of *ESR1* mutations is one of the most common mechanisms of endocrine resistance (14, 35). Since it has been shown that the resistance to TAM is a partial resistance that could be overcome by increasing therapeutic dosage, we will concentrate on the implications of *ESR1* mutations in the resistance to AIs and FUL (36, 37).

## *ESR1* Mutations and AIs

Aromatase is an enzyme of the cytochrome P450 family. This kind of enzyme catalyzes the conversion process of adrenal substrate androstenedione to estrogen in peripheral tissues such as the breast and liver. The rationale for using AIs in post-menopausal patients is to block the production of estrogen by inhibiting aromatase in the tumor and peripheral tissues (32). Patients develop *ESR1* mutations primarily due to the estrogen deprivation therapy including AIs and oophorectomy (38). These mutations are frequently selected in therapy with AIs in metastatic settings, but are rarely acquired in adjuvant therapy with AIs (39). It was shown that detecting circulating *ESR1* mutations at the end of AIs-based adjuvant therapy had no clinical significance, while these mutations could be monitored from first relapse to progression to guide interventional studies (15).

Major genotypic and phenotypic heterogeneity exists among AIs-resistant diseases. Plenty of potential mechanisms have been reported in animal models, but to date the only mechanisms of resistance to ET reported in the clinical practice are HER2 gene amplification and *ESR1* mutations (38, 40). After being constitutively activated by *ESR1* mutations, the ER becomes active without binding to its ligand, conferring complete or partial resistance to AIs (41). This constitutively ligand-independent ERα activity could be explained by a shift in helix 12 within the LBD, which leads to a resemblance to the ligand-bound active state (42). Importantly, numerous studies have identified the prognostic role of *ESR1* mutations in MBC patients who received AIs 17, 27, 39, 43, 44). The rate of *ESR1* mutations in ER+ MBC patients who progressed on AIs treatment was significantly higher than that of AIs-naïve patients (25.8% vs. 0%; *P* = 0.015) (43). Li et al. demonstrated that the change of MAF in circulating *ESR1* was an important biomarker in ER+ MBC, which could predict the resistance to AIs (44). However, the predictive role of *ESR1* mutations in endocrine resistance needs to be validated in further high-quality studies.

## *ESR1* Mutations and FUL

As the only SERD approved currently for clinical practice, FUL (ICI182780) has 100 times higher affinity to the ER than TAM (3, 45). Pre-clinical and clinical data showed that FUL was active even in TAM-resistant models (46, 47)). American Society of Clinical Oncology has recommended FUL 500 mg to be the standard treatment for post-menopausal ER+ ABC patients who fail in the first-line ET (48). In 2016, the FALCON study further proved FUL as an effective first-line ET for post-menopausal HR+ ABC patients (49). Recent data from one real-world study indicated that FUL users with shorter PFS had a significantly higher mutation rate of *ESR1* (0/6 vs. 6/10; Fisher’s exact *P* = 0.03) (50). Different *ESR1* sub-clones *in vitro* may contribute to the differing response to FUL (51). *ESR1* Y537S mutants required the highest dose to completely block transcriptional activity and cell proliferation compared with other mutants (52). The selection of *ESR1* Y537S was identified as the variant that most likely promoted the resistance to FUL (19, 53). Even though the biochemical mechanism of the FUL relative resistance has not been elucidated, patients with mutations in the *ESR1* LBD may benefit from higher-dose or more potent SERMs/SERDs (6, 14, 33).

## Advances in Liquid Biopsy Technologies for Detecting *ESR1* Mutations

Traditional tissue biopsy has high detection rate and accuracy, but it would take a month or later to obtain results. Repeated invasive operations can cause significant harm to the patient and increase staff burden (54). In contrast, liquid biopsy is non-invasive, rapid, and can be conducted in patients with tumors that are inconvenient for biopsy. Nevertheless, the detection rate and sensitivity of liquid biopsy could be low as the CTCs are rare in the blood (≤ 1/ml) and the presence of background DNA (55, 56). Using ultra-deep sequencing to increase detection sensitivity could be unaffordable. Fortunately, the cost of sequencing has been gradually decreasing with the advances of sequencing technology, thus improving its clinical feasibility.

The two most commonly used methods for liquid biopsy in MBC are droplet digital PCR (ddPCR) and NGS. The ddPCR has long been preferred to detect *ESR1* mutations for its higher sensitivity and accuracy (57). Whereas, NGS can interrogate larger regions such as gene panels without prior knowledge of the mutations (19, 58, 59). Several common commercially available NGS technologies include Guardant360 (Guardant Health), FoundationOne Liquid and FoundationACT (Foundation Medicine Inc). Guardant360 is the first analysis tool that combines liquid biopsy and NGS. It can analyze 73 genes of ctDNA in blood samples to guide treatment decisions in seven days (60). FoundationOne Liquid is the only FDA-approved blood-based test to analyze over 300 genes with high accuracy. This test also reports blood tumor mutational burden, microsatellite instability, and tumor fraction values (61). As the predecessor assay of FoundationOne Liquid, FoundationACT detects substitutions, indels, copy number amplifications, and rearrangements in 62 genes and targets ~141 kbp of the human genome (62). As there are certain technical differences between these technologies, researchers must know the scope of detected genes and consult with the company before starting sequencing (63). Recently, substantial effort has been invested in improving the sequencing technologies of liquid biopsy (31, 64–69). The main features of emerging detection technologies such as eTAm-Seq and PredicinePLUS™ were summarized in **Table 2**. Despite the advances of sequencing technologies, there remain some issues to address, including the increasing proportions of false positives and negatives at low MAF and the reproducibility of high throughput technologies (70, 71).

**Table 2:** Selected studies of emerging detection technologies for *ESR1* mutations in MBC.

Author	Year	Study type	Detection technology	Sensitivity	Detection site	Prevalence rate of mutations
Lupini et al. (65)	2018	Retrospective study	NGS or ddPCR based on enhanced-ice-COLD-PCR	0.01%	Codons 536-538	26.8% (15/56)
Fribbens et al. (66)	2018	Prospective study	ddPCR and enhanced tagged-amplicon sequencing (eTAm-Seq)	0.04 to 3.2%	Codons 380, 463, and 536-538	37.5% (27/72)
Masunaga et al. (69)	2018	Observational study	Molecular barcode-NGS	0.1%	Hotspot segment (c.1600–1713) within the *ESR1* LBD	29.4% (10/34)
Ross et al. (67)	2019	Observational study	Memorial Sloan Kettering-Integrated Mutation Profiling of Actionable Cancer Targets	2% for hotspot mutations and 5% for non-hotspot mutations	The whole *ESR1* LBD	11.3% (66/586)
Davis et al. (68)	2019	Observational study	PredicinePLUS™	0.25% for all genomic regions and 0.1% for hotspot variants	180 genes	NA
Jeannot et al. (31)	2020	Retrospective study	Multiplex ddPCR combined with a drop-off assay	0.07 to 0.19%	Codons 380 and 536-538	28.8 (17/59)
Masunaga et al. (64)	2020	Observational study	Molecular barcode-NGS	0.1%	The whole *ESR1* LBD	24.1% (13/54)

MBC, metastatic breast cancer; ddPCR, droplet digital Polymerase Chain Reaction; NGS, Next-Generation Sequencing; LBD, ligand-binding domain.

## Prognostic Role of *ESR1* Mutations in ER+ MBC

ERα-positivity is a favorable prognostic factor for BC, nevertheless, ER+ BC patients tend to lose ERα-positivity as a result of temporal and spatial heterogeneity and can recur several years after the completion of adjuvant therapy (35, 72). *ESR1* mutations accumulate during treatment and may generate a more aggressive phenotype *via* transcriptional changes (16, 27). Many studies have demonstrated that circulating *ESR1* mutations may be poor prognostic factors for ER+ MBC (27, 39, 50, 73–76). A recent meta-analysis demonstrated that plasma *ESR1* mutations carriers had significantly worse progression-free survival (PFS) and OS (*P* < 0.0001) compared with wild-type *ESR1* (75). In AIs-treated MBC patients, *ESR1* mutations carriers also showed significantly shorter PFS than non-carriers (*P* = 0.017) (76). The prognostic value of *ESR1* mutations may be more significant in patients receiving second-line and above ET because of the much higher mutation rates, compared with patients receiving first-line ET (27–30). The cfDNA *ESR1* mutations were associated with shorter overall survival (OS) of MBC patients receiving second-line and above ET from the BOLERO-2 study (27). In real world data, *ESR1* mutations were also found to be related to poor PFS in higher-line fulvestrant (FUL) users (50). However, the *ESR1* ctDNA dynamics in advanced BC (ABC) patients from the PALOMA-3 study offered limited prediction of clinical outcomes (77). This could be explained by the frequent sub-clones of *ESR1*, which will be further discussed in a later section.

## Longitudinal Monitoring and Sub-Clones

Given that cancer is an evolving process based on the clonal theory of tumor evolution, it is somewhat one-sided to only link genomic profile at a certain time point to the prognosis (78). In contrast, longitudinal monitoring of disease may provide more comprehensive information of prognosis and disease progression (44, 79, 80). Patients with persistently elevated CTCs/ctDNA with *ESR1* LBD mutations showed shorter PFS than patients who did not (*P* = 0.0007), while baseline *ESR1* LBD mutation status was not prognostic (79). Dynamically monitoring ctDNA *ESR1* mutations may predict the resistance to AIs (44). Furthermore, circulating *ESR1* variants were shown to be more sensitive in monitoring the evolution and predicting potential resistance than contemporary staging methods (80).

With the genomic profile changing constantly during disease evolution, sub-clones with much aggressive biological behaviors and acquired drug resistance may be selected under the pressure of multiple-line treatment (81). Recent progress suggests that the tumor sub-clones are involved in the metastatic progression and chemotherapy resistance (82). In ER+ MBC patients, detection of CTCs revealed high levels of intra- and inter-tumor heterogeneity (83). Takeshita et al. found that 72.7% (8/11) MBC patients with cfDNA *ESR1* mutations had the poly-clonal mutations during the treatment course, suggesting the differential response of individual *ESR1* mutations to treatments (84). Another study by the same team detected distinct populations of *ESR1* mutations in plasma and corresponding metastatic tissues. Each *ESR1* mutation could have unique clinical significance, and it would be intriguing to investigate them all (19, 85). O’Leary et al. sequenced the ctDNA samples of patients from the PALOMA-3 study. The results showed that 28.9% (33/114) baseline *ESR1* mutations were poly-clonal with the majority of poly-clonal samples featuring a D538G mutation (87.9%, 29/33) (77). D538G mutants demonstrated an enhanced estrogen-dependent response in ER+ T47D cell models, while Y537S mutants showed no estrogen dependence (86). Therefore, longitudinal analysis of cfDNA may help optimizing therapeutic regimens for patients with dominant D538G *ESR1*-expressing clones. Moreover, given that clonal selection of hotspot *ESR1* mutations can occur at the early stage of disease, assessing circulating *ESR1* mutations as soon as first relapse happens may help to guide clinical interventions (15, 76).

## The Crosstalk Between Estrogen and Other Signaling Pathways

Interactions between ER and GFR signaling such as *IGF1R*, HER2, and *FGFR* have been described, suggesting an additional therapeutic strategy to block *ESR1* mutant-driven BC by targeting non-genomic signaling pathways (25, 35, 87–90). *IGF1R* signaling was upregulated in *ESR1* mutant over-expression models and involved in the resistance to TAM (87). *IGF1R* inhibitor (OSI906) and FUL were shown to induce synergistic growth inhibition in Y537S and D538G *ESR1* mutant BC cells (88). HER2, a well-established therapy target in BC, has been shown to phosphorylate and increase ER transcriptional activity when aberrantly activated (89). HER2 amplification was correlated with reduced ER expression, reduced sensitivity to ER targeted therapies, and poor outcomes (91). Toy et al. identified an *ESR1* mutation rate of 7.9% (10/126) in ER+ HER2+ patients, suggesting a potential role of *ESR1* mutations in HER2+ patients. Therefore, it would be necessary to further determine the differences in the treatment outcomes between HER2+ patients with or without *ESR1* mutations (52). *FGFR*, the receptor that binds to members of the fibroblast growth factor family, regulates a series of physiologic cellular processes (90). The deregulation of *FGFR* signaling has been observed in a subset of cancers (92). Formisano et al. identified *FGFR1* signaling as a mechanism of drug resistance in ER+ MCF-7 cells treated with the CDK4/6 inhibitor ribociclib plus FUL. In addition, the presence of ctDNA *FGFR1* alterations was associated with a shorter PFS in the MONALEESA-2 study, suggesting that aberrant *FGFR* signaling was a potential mechanism of escape from ET plus CDK4/6 inhibitors (19, 93).

On the other hand, the interactions between ER and GFR can activate downstream signaling components such as *PI3K*/*AKT*/*mTOR* pathways (94). The ER and *PI3K* pathways often play a synergistic role in tumor progression (95). More ER+ MBC patients with progressive disease exhibited increased *PIK3CA* mutation frequency than those without progressive disease (56.25% vs. 0%; *P* = 0.002). In addition, *GATA3* and *ESR1* mutations correlated with *PIK3CA* mutation, and the combination of everolimus (an *mTOR* inhibitor) and chemotherapy effectively suppressed these gene mutations (96). The *NOTCH* signaling pathway, a highly conserved cell signaling system, includes four different receptors (*NOTCH1*/*2*/*3*/*4*) in mammals (97). Elevated *NOTCH* expression was correlated with poor survival among BC patients (98, 99). High expression of *NOTCH* target genes was exhibited in *ESR1* mutants and blocking *NOTCH* signaling could reduce mammosphere-forming efficiency and migratory potential. These findings warrant further investigation of treatment targeted at *NOTCH* pathway in *ESR1* mutant BC (100).

## Pre-Clinical Studies on Novel SERDs/SERMs

Enhanced ET could potentially overcome resistance caused by the activated somatic mutants and other mechanisms (101). Although FUL has displayed clinical benefit in ABC patients despite its inconvenience and poor bioavailability of intramuscular injections, the drug-resistant phenotypes of the *ESR1* mutants underline the need to develop more potent SERDs/SERMs that are also orally bioavailable (102). GLL398, a boron-modified GW5638 analog, has shown superior ER degrading efficacy and oral bioavailability (103, 104). Compound ERD-148 is a novel orally bioavailable degrader of ERα. ERD-148 inhibited the growth of ER+ BC cells *via* specifically antagonizing the ERα receptor with comparable potency to FUL and marginal non-specific toxicity (105).. Bazedoxifene is a potent anti-estrogen agent that is clinically approved for hormone replacement therapy. Fanning et al. found that bazedoxifene possessed improved inhibitory potency against the D538G and Y537S mutants compared with TAM and had additional inhibitory activity when combined with a CDK4/6 inhibitor palbociclib. Elacestrant, a novel oral SERD, presented growth inhibition in cells and patient-derived xenograft models resistant to all three approved CDK4/6 inhibitors, providing a scientific rationale for evaluating elacestrant in patients pre-treated with CDK4/6 inhibitors (106). Several ongoing clinical trials involving potential novel SERDs were presented in **Table 3**.

**Table 3:** Ongoing clinical studies of novel SERDs in ER+ MBC patients (registered in clinicaltrials.gov until 13 July 2020).

NCT Number	Status	Agent	Condition	Phase	Enrollment	Start Date	Completion Date
NCT02734615	Active, not recruiting	LSZ102	Advanced or metastatic ER+ breast cancer	1	420	June 14, 2016	October 30, 2020
NCT04191382	Recruiting	SAR439859	ER+ HER2- breast cancer	2	126	February 4, 2020	December 2020
NCT04059484	Recruiting	SAR439859	Locally advanced or metastatic ER+ breast cancer	2	372	October 22, 2019	March 2025
NCT03455270	Active, not recruiting	G1T48	ER+ HER2- advanced breast cancer	1	184	May 9, 2018	March 2024
NCT03616587	Recruiting	AZD9833	Advanced ER+ HER2- breast cancer	1	182	October 11, 2018	April 12, 2022
NCT04214288	Recruiting	AZD9833	Advanced ER+ HER2- breast cancer	2	288	April 22, 2020	January 4, 2023

SERDs, selective estrogen receptor down-regulators; ER, estrogen receptor; MBC, metastatic breast cancer; HER2, human epidermal growth factor receptor 2; +, positive; -, negative.

## Conclusions and Prospects

The sequencing technology has been developing towards higher sensitivity and accuracy in recent years. Taking advantage of the advances in liquid biopsy, we can reveal more comprehensive genomic landscape of cancer patients, as well as the potential relationship between genomic alterations and disease progression that is helpful in improving treatment decision-making. Numerous clinical studies based on liquid biopsy have identified the prognostic role of *ESR1* mutations in ER+ MBC, while monitoring *ESR1* mutations alone has not been clinically used for treatment prediction yet. Importantly, *ESR1* mutations may play a key role in the resistance to ET and several potential mechanisms were proposed by some pre-clinical studies. For example, the constitutive activity of ER led by *ESR1* mutations may contribute to resistance to AIs and *ESR1* Y537S sub-clone could be the variant most likely promoting resistance to FUL. In general, large-scale prospective trials are desperately needed to advance the clinical management of *ESR1* mutations in MBC.

Frequently-acquired *ESR1* mutations alongside enhanced estrogen-regulated genes expression, intractable drug resistance, and the defects of existing drugs collectively provide a strong rationale to develop more potent SERMs/SERDs. Moreover, the crosstalk between *ESR1* and other signaling pathways, such as *IGF1R* and *NOTCH*, may offer additional therapy targets and chances to improve survival for patients who have developed ET resistance. Despite encouraging results of the combination of CDK4/6 inhibitors and ET being obtained in previous large-scale clinical studies such as studies of PALOMA series and MONALEESA series, many questions remain unaddressed. For example, can *ESR1* mutations in ER+ MBC patients be reversed due to the effective targeted therapy? Or will there be differences in PFS and OS between *ESR1* mutations carriers and non-carriers in the setting of CDK4/6 inhibitors plus ET? Future studies should focus on developing novel sequencing technologies to further unveil the biological and clinical implications of *ESR1* mutations in ER+ MBC, especially the relationships between each *ESR1* sub-clone and endocrine resistance.

## Author Contributions

All authors listed have made a substantial, direct, and intellectual contribution to the work and approved it for publication.

## Conflict of Interest

The authors declare that the research was conducted in the absence of any commercial or financial relationships that could be construed as a potential conflict of interest.
